# Supplementary material for: Effects of Soybean Phosphate Transporter Gene GmPHT2 on Pi Transport and Plant Growth under Limited Pi Supply Condition
Source: Int J Mol Sci. 2023 Jul 5;24(13):11115. doi: 10.3390/ijms241311115 (PMC10342517; doi:10.3390/ijms241311115)
Supplement: Supplementary file 1 [file ijms-24-11115-s001.zip › FigureS1.pptx]

## Slide 1
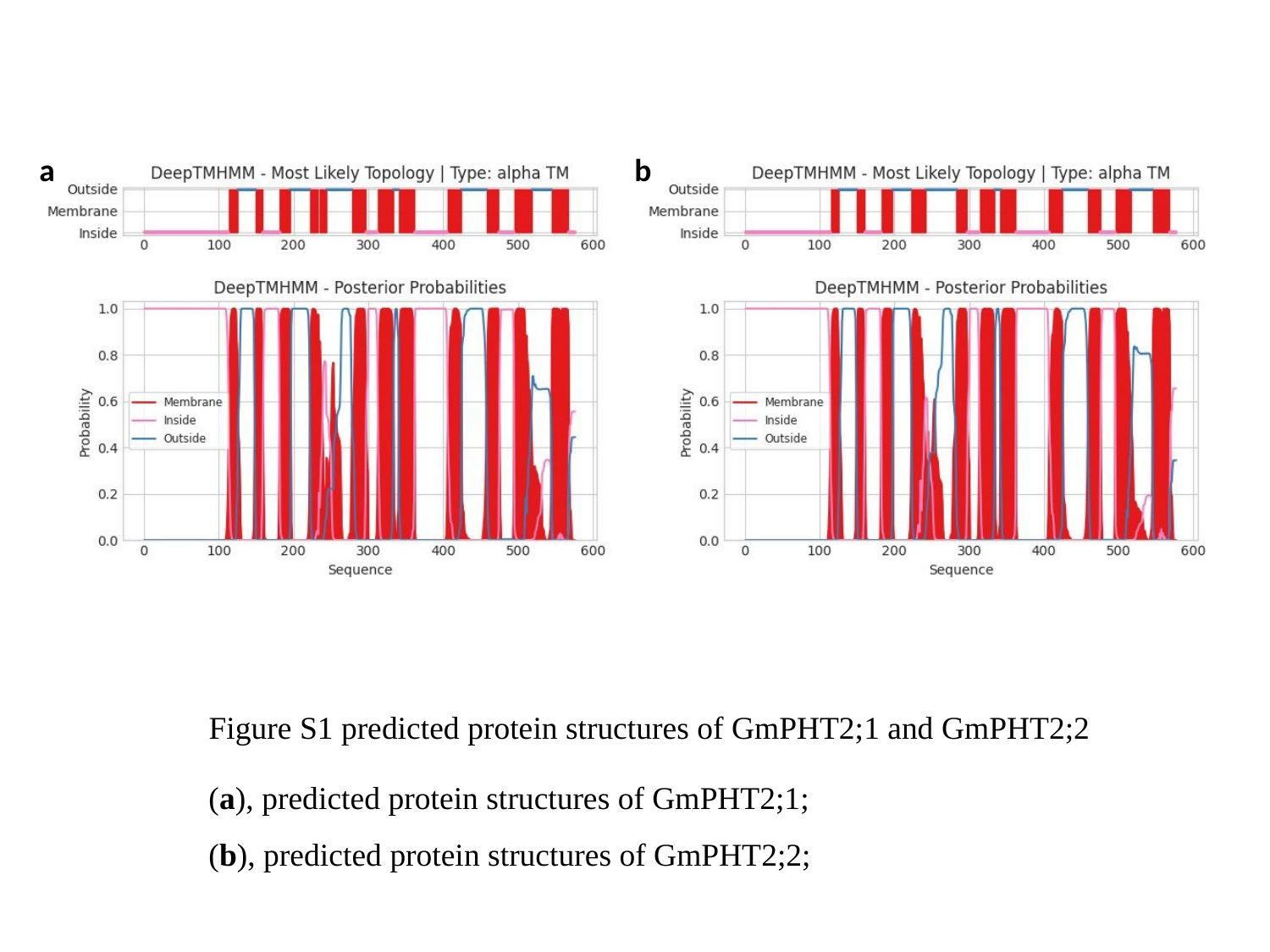

a
b
Figure S1 predicted protein structures of GmPHT2;1 and GmPHT2;2
(a), predicted protein structures of GmPHT2;1;
(b), predicted protein structures of GmPHT2;2;
