# Supplementary material for: Effects of Soybean Phosphate Transporter Gene GmPHT2 on Pi Transport and Plant Growth under Limited Pi Supply Condition
Source: Int J Mol Sci. 2023 Jul 5;24(13):11115. doi: 10.3390/ijms241311115 (PMC10342517; doi:10.3390/ijms241311115)
Supplement: Supplementary file 1 [file ijms-24-11115-s001.zip › Table S1.pdf]

Table S1. PHT2 family's genes of 49 plant species.

Number	Organism	Gene ID	Protein Length	Number of exons
1	Ricinus communis	27504.m000647	476	3
2	Triticum aestivum L.	AF156696	537	3
3	Capsicum annuum L.	AF533081	574	3
4	Arabidopsis lyrata	AL5G15360.t1	586	3
5	Aquilegia coerulea	Aqcoe3G144600.1	579	3
6	Arabidopsis thaliana columbia	AT3G26570.1	613	4
7	Solanum melongena L.	AY293827	568	3
8	Boechera stricta	Bostr.0556s0327.1	586	3
9	Brachypodium distachyon	Bradi3g47550.1	569	3
10	Brachypodium stacei	Brast04G151800.1	569	3
11	Capsella grandiflora	Cagra.3598s0004.1	586	3
12	Capsella rubella	Carubv10016883m	601	4
13	Citrus clementina	Ciclev10031076m	575	3
14	Daucus carota	DCAR_026513	580	3
15	Medicago	EF094558	550	3
16	Carica papaya	evm.model.supercontig_33.20	586	3
17	Gossypium raimondii	Gorai.005G178500.1	585	3
18	Zea mays Ensembl-18	GRMZM2G092780_T01	577	3
19	Vitis vinifera Genoscope.12X	GSVIVT01005753001	388	4
20	Kalanchoe fedtschenkoi	Kaladp0034s0076.1	579	3

21	Kalanchoe laxiflora	Kalax.0023s0132.1	579	3
22	Oryza sativa	LOC_Os02g38020.1	572	3
23	Manihot esculenta	Manes.12G023200.1	579	3
24	Marchantia polymorpha	Mapoly0138s0013.1	613	3
25	Medicago truncatula	Medtr8g069390.1	574	3
26	Mimulus guttatus	Migut.K00598.1	583	3
27	Citrus sinensis	orange1.1g012038m	472	3
28	Panicum hallii	Pahal.A02359.1	571	3
29	Panicum virgatum	Pavir.Aa01470.1	614	3
30	Solanum tuberosum	PGSC0003DMT400047869	581	3
31	Phaseolus vulgaris	Phvul.003G064900.1	568	3
32	Physcomitrella patens	Pp3c9_7350V3.1	589	3
33	Prunus persica	Prupe.1G244400.1	589	3
34	Salix purpurea	SapurV1A.0317s0260.1	587	3
35	Setaria italica	Seita.1G216200.1	572	3
36	Setaria viridis	Sevir.1G220000.1	572	3
37	Sphagnum fallax	Sphfalx0052s0101.1	604	3
38	Theobroma cacao	Thecc1EG010516t1	585	3
39	Eutrema salsugineum	Thhalv10003891m	586	3
40	Trifolium pratense	Tp57577_TGAC_v2_mRNA18371	583	3
41	Zea mays PH207	Zm00008a022208	577	3
42	Brassica oleracea capitata	Bol032193	573	3
43	Brassica oleracea capitata	Bol042818	581	3

44	Brassica rapa FPsc	Brara.F03266.1	586	3
45	Brassica rapa FPsc	Brara.I00256.1	573	3
46	Eucalyptus grandis	Eucgr.B03285.1	475	3
47	Eucalyptus grandis	Eucgr.H02499.1	572	3
48	Glycine max	Glyma.08G282100	575	3
49	Glycine max	Glyma.18G144100	576	3
50	Sorghum bicolor	Sobic.004G199900.1	572	3
51	Sorghum bicolor	Sobic.004G199900.1	577	3
52	Populus trichocarpa	Potri.008G186600.1	420	3
53	Populus trichocarpa	Potri.010G046300.1	590	3
