# Supplementary material for: Effects of Soybean Phosphate Transporter Gene GmPHT2 on Pi Transport and Plant Growth under Limited Pi Supply Condition
Source: Int J Mol Sci. 2023 Jul 5;24(13):11115. doi: 10.3390/ijms241311115 (PMC10342517; doi:10.3390/ijms241311115)
Supplement: Supplementary file 1 [file ijms-24-11115-s001.zip › Table S2.pdf]

Table S2. 52 PHT2 family protein sequences from 49 species.

>PcPHT2.1
MAKAFNISSRTASAIAIFIAFAALSLPLFMKSLGTGLDLKTKILSYATLLFGFYMAWNIGANDVANAM
GTSVGSGALSLRQAVVTAAILEFSGAFLMGTHVTNTMQNGILMANVFEGKNTLLFAGLLSSLAAA
GTWLQVASYYGWPVSTTHCIVGSMVGFGLVYGGAGAVFWSSLARVTSSWVISPVMGAMVSFLV
YKCIRRFVYSAPNPGQAAAAAAPIAVFAGVTGISFAAFPLSKTFPVALVQALACGAAGAFLVYRIIHK
QLGHLLVKANTTQPEPKESTIQNKNIGFLSDIAGPTGTQLEIVYGVFGYMQVLSACFMSFAHGGND
VSNAIGPLAAALSILHGGASGTEIVIPTDVLAWGGFGIVAGLTMWGYRVIATIGKKITELTPTRGFAA
EFAAASVVLVASKLGLPISATHTLVGAVMGVGFARGLNSVRAETVREIVASWLVTIPAGATFAVLYT
WILTKLLSSLL
>TaPHT2.1
MTLPYRFSSVRNHSLLLKTSHLYTPRSALGCFSPKESPFFKKNTAQFLSPQKHTSLPLKLVCPLASFSS
YADSEEEEQHHADQPIQNSHESSTVSSGSDGKGNAEATGDFSGMAQAFHISSTTARAISIVIAFSAL
TLPIFMKSLGQGLALKTKLLSYATLLFGFYMAWNIGANDVANAMGTSVGSGALTIRQAVMTAAVL
EFSGALLMGTHVTSTMQKGILMANVFQGKDMLLFAGLLSSLAAAGTWLQVASYYGWPVSTTHCI
VGSMVGFGLVYGGAGAVFWSSLAKVASSWVISPLLGALVSFLVYKCIRRFVYSAPNPGQAAAAAA
PVAVFVGVASISSAALPLSKIFPIALSQALACGVAGAIVFDRIIRKQLGHLLAKTKSPETSQNQPKTIGF
LSDIAGPTGTQLEIVYGIFGYMQVLSACFMSFAHGGNDVSNAIGPLAAALSILQNGAAAGGAEIVIP
MDVLAWGGFGIVAGLTMWGYRVIATIGKKITELTPTRGFAAEFAAASVVLFASKLGLPISATHTLVG
AVMGVGFARGLNSVRAETVREIVASWLVTIPVGATLSVIYTWIFTKILSFVL
>AcPHT2.1
MTPSHCFSSSIRRIGGGAEAFLLHKQISSHFYYDTHIFKRDDKTLQLKSQPLKSPLLPILRLRSLKPLLPS
ASLSSFAEPEGEEQEVIIDKKQEDGESDDLPGMAQAFHISSRTATAIIFCIAVAALSFPFVMKSMGQG
MGLKNKILSYTTLLFGFYMAWNIGANDVANAMGTSVGSGALSLRQAVLTAAVLEFSGALLMGTH
VTSTMQKGILVANVFQGKDSLLFTGLLSSLAAAGTWLQVASYYGWPVSTTHCIVGAMVGFGLVY
GGFGAVFWSSLARVTASWVISPFMGAAVSFIVYKCIRRFVYSAPNPGQAAAAAAPVAVFVGVTAIS
FAAFPLSKIVPVAVAQALACGTVGAILVSRVIHKQLGHLLLKSNSSQLETNEDASQNKGIGLLSDVA
GPKGTQLKIVYGVFGYMQVLSACFMSFAHGGNDVSNAIGPLAAALSILHGGASGAEIVIPIDVLAW
GGFGIVAGLMIWGYRVIATIGKKITELTPTRGFAAEFAAASVVLFASKLGLPISATHTLVGAVMGVGF
ARGLNSVRAETVKEIVTSWAITIPIGAILAVFYTWILKRLLSGLL
>AtPHT2.1
MQIHFHHHQYHHQLHSHHPWPISHLYMTLPYRFSSVRNHSLLLKTSHLCTPRSALGCCFSPKESPF
FRKNTAQFLSPQKHTSLPLKLVCPLASFSSYADSEGEEQHHADQPIQNPHESSTVSNESDGKGNAE
ATGDFSGMAQAFHISSTTARAISIVIAFSALTLPIFMKSLGQGLALKTKLLSYATLLFGFYMAWNIGA
NDVANAMGTSVGSGALTIRQAVMTAAVLEFSGALLMGTHVTSTMQKGILMANVFQGKDMLLFA
GLLSSLAAAGTWLQVASYYGWPVSTTHCIVGSMVGFGLVYGGAGAVFWSSLAKVASSWVISPILG
ALVSFLVYKCIRRFVYSAPNPGQAAAAAAPVAVFVGVASISSAALPLSKIFPIALSQALACGVAGAIV
FDRIIRKQLGHLLAKTKSPETSQNQPKTIGFLSDIAGPTGTQLEIVYGIFGYMQVLSACFMSFAHGGN
DVSNAIGPLAAALSILQNGAAAGGAEIVIPMDVLAWGGFGIVAGLTMWGYRVIATIGKKITELTPTR
GFAAEFAAASVVLFASKLGLPISATHTLVGAVMGVGFARGLNSVRAETVREIVASWLVTIPVGATLA
VIYTWIFTKILSFVL
>BoPHT2.1
MTLPYGLSSVRNHSLLLKTSHLCVPRSALGCFSPKESPLFKKNSALFLSPQKHTSFPLKLVHPLASFSS
YAEGDGDQQIQNSHDSDEKGNAEAVGDFSGMANAFHISSKTARAITIVIAFSALSLPLFMKSLGQG

LALKTKLLSYATLLFGFYMAWNIGANDVANAMGTSVGSGALTIRQAVITAAVLEFSGALLMGTHVT
STMQKGILMANVFQGKDMLLFAGLLSSLAAAGTWLQVASYFGWPVSTTHCIVGSMVGFGLVYG
GAGAVFWSSLAKVASSWVISPLMGALVSFLVYKCIRRFVYSAPNPGQAAATAAPAAVFLGVASISF
AAFPLSKIFPIALSQALACGAAGAIIFDRIIRDKLGHLLAKTKPLDTPQNQPKSIGFLSDIAGPTDTQLEI
VYGVFGYMQVLSACFMSFAHGGSDVSNAIGPLAAALSILQRGATGGGGDIVIPMDVLAWGGFGI
VAGLTMWGYRVIATIGKKITELTPTRGFAAEFAAASVVLFASKLGLPISATHTLVGAVMGVGFARGL
NSVRAETVKEIVASWLVTIPVGGTLAVIYTWVFTKLLSFVL
>BoPHT2.2
MTLPYGFSSVRNHSLMLKTSHLCVPRSAIGCFSPKESPFFKKNSAIFLSPQKHTKLVCPLASFSSYAE
GDEQHHGDQQIQNSHDSSTNSNESDGKDNPEATGDFSGMANAFNISSKTARAITIVIAFSALSLP
LFMKSLGQGLARKTKILSYATLLFGFYMAWNIGANDVANAMGTSVGSGALTIRQAVITAAVLEFSG
ALLMGTHVTSTMQKGILMANVFQGKDMLLFAGLLSSLAAAGTWLQVASYYGWPVSTTHCIVGS
MVGFGLVYGGAGAVFWSSLAKVASSWVISPVMGALVSFLVYKCIRRFVYSAPNPGQAAAAAAPV
AVFVGIASISSAAFPLSKTFPIALSQALACGAAGAIIFDRIIRNKLGHLLAKTKPLDTAQTQPKDIGFLS
EIAGPTGTQLKIVYGVFGYMQVLSACFMSFAHGGNDVSNAIGPLAAALSILQRGAGAGGGGEIVIP
IDVLAWGGFGIVAGLTMWGYRVIATIGKKITELTPTRGFAAEFAAATVVLFASKLGLPISATHTLVGA
VMGVGFARGLNSVRAETVKEIVASWLVTIPVGATLAVVYTWVFTKILSFVL
>BsPHT2.1
MTLPYRFSSVRNHSLLLKTSHLCTPRSALGCFSPKESPLFKKNTAQFLSPQKHTSLPLKLVCPLASFSS
YADYEGDEQHHADKPIQNSHESSTDPSGSDGKGNAEATGEFSGMAEAFHISSTTARAISIVIAFSAL
TLPLFMKSLGQGLALKTKLLSYATLLFGFYMAWNIGANDVANAMGTSVGSGALTIRQAVMTAAVL
EFSGALLMGTHVTSTMQKGIIMANVFQGKDMLLFAGLLSSLAAAGTWLQVASYYGWPVSTTHCI
VGSMVGFGLVYGGAGAVFWSSLAKVASSWVISPLLGALVSFLVYKCIRRFVYSAPNPGQAAAAAA
PVAVFVGVTCISSAALPLSKIFPIALSQALACGVAGAIVFDRIIRKQLGHLLAKTKTPETSQNQPKTIGF
LSDIAGPTGTQLEIVYGIFGYMQVLSACFMSFAHGGNDVSNAIGPLAAALSILQSGAAAGGGEIVIP
MDVLAWGGFGIVAGLTMWGYRVIATIGKKITELTPTRGFAAEFAAASVVLFASKLGLPISATHTLVG
AVMGVGFARGLNSVRAETVREIVASWLVTIPVGGTLAVIYTWIFTKILSFVL
>BdPHT2.1
MSQSSPFFSIARAHAGAGGRAAAAALLLRHPAAQLPPSIHCPRYFPLAKLSHAKPLNSHLGFPRATL
SSFADANDGSSAEPDASEEQNGGSELSEMAKAFHISPRTAMSISVVIAFAALTVPLAMRSLAFHGTI
KMKMLAYLTLLSGFYMAWNIGANDVANAMGTSVGSGALTLRQAVLTAAVLEFSGAFLMGTHVT
STMQKGILVASVFQGNDSLLFAGLLSSLAAAGTWLQVASSYGWPVSTTHCIVGAMVGFGLVYGG
VNAVFWSSLARVSSSWVISPLMGAAVSFLVYKCIRRFVYSAPNPGQAAAAAAPIAVFTGVTAISFA
AFPLSKIFSIAVLQALGCGAIGAIFVSRAIKKQLGDLLSSEAEKIASASNTDGQQGGFLSDVAGPTGA
QLQIVYGVFGYMQVLSACFMSFAHGGNDVSNAIGPLAAALSLLQGVATSAEIVIPTEVLAWGGFGI
VAGLTMWGYRVIATIGKKITELTPTRGFAAEFAAASVVLFASKLGLPISATHTLVGAVMGVGFARGL
NRVRAETVREIVVSWVVTIPVGALLAVFYTLILTKILKYFM
>BrPHT2.1
MTLPYGFSSVRNHSLMLKTSHLCVPRSAIGCFSPKESPFFKKNSSRFLSPQKHTSLPLKLVCPLASFSS
YADGDEQHHGDQQIQNSHNSSTNSNESDGKGNPEATGDFSGMANAFNISSKTARAITIVIAFSAL
SLPLFMKSLGQGLALKTKILSYATLLFGFYMAWNIGANDVANAMGTSVGSGALTIRQAVITAAVLE
FSGALLMGTHVTSTMQKGILMANVFQGKDMLLFAGLLSSLAAAGTWLQVASYYGWPVSTTHCIV
GSMVGFGLVYGGAGAVFWSSLAKVASSWVISPVMGALVSFLVYKCIRRFVYSAPNPGQAAAAAA
PVAVFVGIASISSAAFPLSKTFPIALSQALACGAAGAIIFDRIIRNKLGHLLAKTKPLDTAQTQPKDIGF

LSEIAGPTGTQLKIVYGVFGYMQVLSACFMSFAHGGNDVSNAIGPLAAALSILQRGAGAGGGGGE
IVIPIDVLAWGGFGIVAGLTMWGYRVIATIGKKITELTPTRGFAAEFAAATVVLFASKLGLPISATHTLV
GAVMGVGFARGLNSVRAETVKEIVASWLVTIPVGATLAVIYTWVFTKILSFVL
>BrPHT2.2
MTLPYGLSSVRNHSLLLKTSHLCVPRSALGRFSPKESPLFKKNSALFLSPQKHTSLPLKLVHPLASFSS
YAEGDGDQQIQNSHDSDEKGNAEAVGDFSGMANAFHISSKTARAITIVIAFSALSLPLFMKSLGQG
LALKTKLLSYATLLFGFYMAWNIGANDVANAMGTSVGSGALTIRQAVITAAVLEFSGALLMGTHVT
STMQKGILMANVFQGKDMLLFAGLLSSLAAAGTWLQVASYFGWPVSTTHCIVGSMVGFGLVYG
GAGAVFWSSLAKVASSWVISPLMGALVSFLVYKCIRRFVYSAPNPGQAAATAAPVAVFLGVASISF
AAFPLSKIFPIALSQALACGAAGAIIFDRIIRDKLGHLLAKTKPQDTPQNQPKSIGFLSEIAGPTDTQLE
IVYGVFGYMQVLSACFMSFAHGGSDVSNAIGPLAAALSILQRGAAGGGGDIVIPMDVLAWGGFGI
VAGLTMWGYRVIATIGKKITELTPTRGFAAEFAAASVVLFASKLGLPISATHTLVGAVMGVGFARGL
NSVRAETVKEIVASWLVTIPVGGTLAVIYTWVFTKLLSFVL
>BtPHT2.1
MSQSSPFFSIARAHAGAGGRAAAAALLLRHPAAQLPPSIHCPRYFPLAKLSHAKPLSSYLGLPRATL
SSFANANDGSGAEPDASEEQNGGSELSEMAKAFHISPRTAMSISVVIAFAALTMPLAMRALAFHG
TIKMKMLAYLTLLSGFYMAWNIGANDVANAMGTSVGSGALTLRQAVLTAAVLEFSGAFLMGTHV
TSTMQKGILVASVFQGNDSLLFAGLLSSLAAAGTWLQVASSYGWPVSTTHCIVGAMVGFGLVYG
GVNAVFWSSLARVSSSWVISPLMGAAVSFLVYKCIRRFVYSAPNPGQAAAAAAPIAVFTGVTAISF
AAFPLSKIISIAVLQALGCGAIGAIFVSRAIKKQLGDLLSSEAEKIASASNTDGQQGGFLSDVAGPTGA
QLQIVYGVFGYMQVLSACFMSFAHGGNDVSNAIGPLAAALSLLQGVATSAEIVIPTEVLAWGGFGI
VAGLTMWGYRVIATIGKKITELTPTRGFAAEFAAASVVLFASKLGLPISATHTLVGAVMGVGFARGL
NRVRAETVREIVVSWVVTIPVGALLAVFYTLILTKILKYFM
>CgPHT2.1
MTLPYRFSSVRNHSLLLKTSHLCTPRSPLGCFSPKESPLFKKNTALFLSPQKHTSLPLKLVCPLASFSSY
ADSEGEEQHHADQPIQNSHESSTDSSGSDGKGNAEATGDFSGMAQAFHISSTTARAISIVIAFSAL
CLPLFMKSLGQGLALKTKLLSYATLLFGFYMAWNIGANDVANAMGTSVGSGALTIRQAVMTAAV
LEFSGALLMGTHVTSTMQKGIIMANVFQGKDMLLFAGLLSSLAAAGTWLQVASYYGWPVSTTHCI
VGSMVGFGLVYGGAGAVFWSSLAKVASSWVISPLLGALVSFLVYKCIRRFVYSAPNPGQAAAAAA
PVAVFVGVASISSVALPLSKIFPIALSQALACGVAGAIVFDRIIRKQLGHLLAKTKSPETSQNTPKAIGF
LSDIAGPTGTQLEIVYGIFGYMQVLSACFMSFAHGGNDVSNAIGPLAAALSILQNGAAAGGAEIVIP
MDVLAWGGFGIVAGLTMWGYRVIATIGKKITELTPTRGFAAEFAAASVVLFASKLGLPISATHTLVG
AVMGVGFARGLNSVRAETVKEIVASWLVTIPVGATLAVIYTWIFTKILAFVL
>CrPHT2.1
MTLPYRFSSVRNHSLLLKTSHLCTPRSALGCFSPKESPLFKKNTALFLSSQKHTSLPLKLVCPLASFSS
YADSEGEEQHHADQPIQNSHESSADSSGSDGKGNAEATGDFSGMAQAFHISSTTARAISIVIAFSA
LCLPLFMKSLGQGLALKTKLLSYATLLFGFYMAWNIGANDVANAMGTSVGSGALTIRQAVMTAA
VLEFSGALLMGTHVTSTMQKGIIMANVFQGKDMLLFAGLLSSLAAAGTWLQVASYYGWPVSTTH
CIVGSMVGFGLVYGGAGAVFWSSLAKVASSWVISPLLGALVSFLVYKCIRRFVYSAPNPGQAAAAA
APVAVFVGVASISSVALPLSKIFPIALSQALACGVAGAIVFDRIIRKQLGHLLAKTKSPETSQNTPKAIG
FLSDIAGPTGTQLEIVYGIFGYMQVLSACFMSFAHGGNDVSNAIGPLAAALSILQNGAAAGGAEIVI
PMDVLAWGGFGIVAGLTMWGYRVIATIGKKITELTPTRGFAAEFAAASVVLFASKLGLPISATHTLV
GAVMGVGFARGLNSVRAETVKEIVASWLVTIPVGATLAVIYTWIFTKILAFVFVLSCRQSKLGDVLLL
>CcPHT2.1

MPPPFCSSSSRNTVSPEAFLFHKHRSSSSALLLKPNLSLPRSSYSLLSLKNSRLTHSFASISSFAEAEGE
EEQNEGLQVQEHHHDEPAKTEAGDDELPGMAQAFHISSSTASAISICIALAALTLPFFMKSLGQGL
DIKTKLLSHATLLFGFYMAWNIGANDVANAMGTSVGSGALTLRQAVLTAAVLEFSGALLMGTHVT
STMQKGILVASVFQGKDTLLFAGLLSSLAAAGTWLQVASYYGWPVSTTHCIVGSMVGFGLVYGGA
GAVFWSSLARVTSSWVISPILGALVSFLVYKCIRRFVYSAPNPGQAAAAAAPIAVFVGVTGISFAAFP
LSKIFPLALAQALAFGAAGAFLVYRIIQKQLGHLLVKSTSLQPEPKDTNIHNKSIGIFSDIAGPKGTQLE
IVYGVFGYMQVLSACFMSFAHGGNDVSNAIGPLAAALSILHGSASGTKIVIPIDVLAWGGFGIVSGL
MIWGYRVIATIGKKITELTPTRGFAAEFAAASVVLFASKLGLPISATHTLVGAVMGVGFARGLNSVR
AETVKEIVLSWAVTIPVGATFAVFYTWILTKLLSFIY
>DcPHT2.1
MTSSYCLSSTRNTVKPRAFLIHSSHFDQSKYRPPFFSLSLKKDHKKLLSSRLYFPTLSAENYDLTRPFA
SLSSFAEADGEENSEKIHALSEHQENEKQQEELQGMAQAFNIPSSTATAISICIALAALCFPYFMNSL
GQGMSLKTKLLSYGTLLFGFYMAWNIGANDVANAMGTSVGSGALTLRQAVVTAAVLEFSGALLM
GKHVTGTIQKGILVADVFQGKDTLLLAGLLSSLAAAGTWLQVASYYGLPVSTTHCIIGSMVGFGLIY
GGRDAVFWGSLARVTSSWLISPVTGAAVSFLVYKCIRRFVYSAPNPGQAAAAAAPIGVFIGVTGISF
IAFPLSKILSVAIVQALALGTVGAFLFDRIIRKQLGHLLVKSVSAQSDPKEDTIHQKNIGLLAEVAGPK
GTQLEIVYKVFGYMQVLSACFMSFAHGGNDVANAIGPLAGALSILQGGTSGGELIIPIDVIAWGGF
GIVAGLMMWGYRVIATIGNKITELTPTRGFAAEFAAASVVMFASNLGLPISATHTLVGAVMGVGFA
RGLNSVRAETVKEIVTSWAVTIPVGAVLAIVYTWILNRLLSYVL
>EgPHT2.1
MAQAFGISSRTASAISICIAFAALLLPLAMRSLGQGMAVKTRALSYGTLLFGFYMAWNIGANDVAN
AMGTSVGSGALTLRQAVLTAAVLEFSGALLMGTHVTSTMQKGILVADVFKGKDTLLFAGLLSSLAS
AGTWLQVASFYGWPVSTTHCIIGSMVGFGLVYGGAGAVFWNSVVRVTSSWVISPLMGALVSFLV
YKCIRRFVYSAPNPGQAAAAAAPIAVFLSVTGISFAAFPLSKTIPLALAQALACGTAGAFLVNRMIRK
QLGHLLDKTDPSQPKPEESMVNRDVGFLSKIAGPTGAQLEIVYGVFGYMQVLSACFMSFAHGGN
DVANAIGPLASALSILQGGATGTDIVIPIDVLAWGGFGIVAGLIMWGYRVIATIGKKITELTPTRGFAA
EFAAASVVLFASKLGLPISATHTLVGAVLGVGFARGLNSVRAETVKEIVVSWAVTIPVGAVFAVFYT
WILTKLLSYIL
>EgPHT2.2
MTSSYFLSSTKQYATTPEPLFLLRRRRHHLPRHRVLSLLSSDIGEAHFPRKPSTFPLQLLRPSHRLGKP
SFATTISSSFEGEEEGEQRQDEVPEGMAEAFHISSSTATAISVLIAVAVFSFPLLMKSQLGAGAPLKSR
ALTYVTLLFGFYMAWNIGANDVANAMGTSVGSGALTLRQAVVTAGVLEFSGALLMGTHVTSTM
QKGILVAGVFQGKDSLLFAGLLSSLAAAGTWLQIASYYGWPVSTTHCIVGSMVGFGLVYGGPGAV
FWTSLARVMSSWVVSPLMGALVSFVVYKCIRRFVYSAPNPGQAAAAAAPVAVFVGVTAISFVAFP
LSKSFPLAVAQALACGTAGAVLVNRIIWKQLGHLLDKAGSSGMEPKEAPALNQNIGFLTEIAGPKG
TQLEIVYGVFGYMQVLSACFMSFAHGGNDVSNAIGPLAGALSILHGGAGGAEIVIPTDVLAWGGF
GIVAGLMMWGYRVIATIGKKITELTPTRGFAAEFAAATVVLFASKLGLPISATHTLVGAVMGVGFAR
GLNSVRAETVREIVASWAVTIPVGAFFAVVYTWILTKLLSYVF
>CpPHT2.1
MTLPYSFSSARNTTTTSPEAFFLHNFHLYLPKHRCYFLCNDTHFRKKDSLLFKYHLNPLHPHLPLLRV
NNCKLTKPFASISSFAEADGGEEQKEGIQIEKQKGEDDDLPGMAQAFHISSSTASAISICIAFAALTLP
FFMKSLAQSLDLRTKLMSYATILLGFYMAWNIGANDVANAMGTSVGSGALTLRQAVVTAAILEFS
GALLMGSHVTGTMQKGILVASVFQGKDTLLFTGLLSSLASAGVWLQVASYYGWPVSTTHCIVGS
MVGFGLVYGGAGAVFWSSLARVTSSWVISPLMGALASFLVYKCIRRFVYSAPNPGQAAAAAAPIA

VFLGVTGISFAAFPLSKNFPVALVQALACGATGAVLVDRIIRKQLGHLLAKSTSSQQETKENTTRHK
NLGLLSDIAGPKGTQLEIVYGVFGYMQVLSACFMSFAHGGNDVSNAIGPLAAALSILHGTSSGAELI
IPTDVLAWGGFGIVAGLMMWGYRVIATIGKKITELTPTRGFAAEFAAASVVLFASKLGLPISATHTLV
GAVMGVGFARGLNSVRSETVREIVASWAVTIPVGATLSVIFTWILTKLLSCLL

>GmPHT2.1

MNPSCRFSSTKHTSFLHNSHTHLSKTTSPYVFLGHSHSQKPFLLSPPITTKPYPFNNILRFRNIKLSYP
FATLSSFAEGEQQENQEGTETPPSNDELGGIAKAFNISSRTASAISICMALAVLTFPLFMTSLGQGM
VLKTKVLSYATLLFGFYMAWNIGANDVANAMGTSVGSGALTLRQAVLTAAVLEFSGALMMGTHV
TSTMQKGILVANVFNGKDSLLFAGLLSSLAAAGTWLQFASYYGWPVSTTHCIVGAMVGFGLAYG
GAGAVFWGSLARVISSWVVSPLMGAAVSFLVYKCIRRFVYSAPNPGLAAAAAAPIAVFLGVTGISF
VAFPLSKSFPFALVQALASGTVGAFLVDRIIRKQLGHLLVKSSTPEPEPKEDTVHHHNIGFLDDVAGP
KGAQLEIVYGVFGYMQVLSACFMSFAHGGNDVSNAIGPLAGALAILQGSTMGTEIIIPTDVLAWG
GFGIVAGLMMWGYRVIATIGKKITELTPTRGFAAEFAAASVVLFASKLGLPISATHTLVGAVMGVGF
ARGFNSVRSETVKEIVASWVVTIPVGASLSVLYTWILTKILSYIL

>GmPHT2.2

MNPSCRFSSTKHTPFLHNSKPTSPFFLRHSHSHSHQVKPFPLSPPITTTTTAKPYPLNILRLRNIKLSH
PLATLSSFAEGEEENQKEGAESPPNNDELGGIAKAFNISSRTASAISICMALAVLTFPLFMTSLGQGM
ALKTKVLSYATLLFGFYMAWNIGANDVANAMGTSVGSGALTLRQAVLTAAVLEFSGALMMGTHV
TSTMQKGILVANVFNGKDSLLFAGLLSSLAAAGTWLQFASYYGWPVSTTHCIVGAMVGFGLAYG
GAGAVFWGSLARVISSWVVSPLMGAAVSFLVYKCIRRFVYSAPNPGLAAAAAAPIAVFLGVTGISF
VAFPLSKNFPLALAQALACGTVGAFLVDRIIRKQLGHLLVKSNTPEPEPKEDSVHHHNIGFLDDVAG
PKGAQLEIVYGVFGYMQVLSACFMSFAHGGNDVSNAIGPLAGALAILQGGAAGTEIVIPTDVLAW
GGFGIVAGLMMWGYRVIATIGKKITELTPTRGFAAEFAAASVVLFASKLGLPISATHTLVGAVMGVG
FARGLNSVRSETVKEIVASWVVTIPVGATLSVLYTWILTKILSYIL

>GrPHT2.1

MTPFSCLPCARSTTSPDAFLLRYPHLRLPKCRHYEHQLPKIENLMFRPFQVPTRPYLPILSLRNSRRT
HPCANISSFAEAEGGKEQNDEAIHLGTRQEDAKPNEEYLPGMAQAFNISSRTASAIAIFIAFAALTLP
FFMKSLGQGVGVKTKFLSYATLLFGFYMAWNIGANDVANAMGTSVGSGALTLRQAVVTAAILEFS
GALLMGTHVTGTMQNGILVANMFQGKDTLLFAGLLSSLASAGTWLQVASYYGWPVSTTHCIVGS
MVGFGLAYGGAGAVFWTSLARVASSWVISPVLGALVSFLVYKCIRRFVYSARNPGQAAAAAAPIA
VFLGVTGISFAAFPLSKSFPIALAQALGCGAVGAVLVYNLIRKQLGHLLEKSASSHSEPQESNTQTKN
LGLLSDVAGPKGTQLEIVYGVFGYMQVLSACFMSFAHGGNDVSNAIGPLAGALSILQGGASSAEIV
IPNDVLAWGGFGIVAGLLIWGYRVIATIGKKITELTPTRGFAAEFAAASVVLFASKLALPISATHTLVG
AVMGVGFARGLNSVRAETVREIVTSWAVTIPVGASLAVLYTWILTKLLSFIL

>ZePHT2.1

MSQSSPLFSVARAHAGAGGRAVAAALLLRRRPIAQLPHSIHGLRCLSPARATPSKTLLSLPRATLSSF
ADPDGDGGSSAKASDGGAKAGAGGEEQVGECEMSEMARAFHISPRVATSISVTIAFAALTVPLAM
RSLVCHGTFRMNALAYFTLLSGFYMAWNIGANDVANAMGTSVGSGALTLRQAVLTAAVLEFSGA
FLMGTHVTSTMQKGILVASAFQGKDSLLFAGLISSLAAAGTWLQVASSYGWPVSTTHCIVGAMVG
FGLVYGGVDAVFWSSLARVSSSWVISPLMGAAVSFLVYKCIRRFVYSAPNPGQAAAAAAPIAVFTG
VTAISFAAFPLSKVFSIAILQAVSSGAIGAIIVSRVIQKQLGDLLSSEAEKIASAEKSSAQQAGFLSDIAG
PTGAQLQIVYGVFGYMQVLSACFMSFAHGGNDVSNAIGPLAAALSILQGVASSAEIVIPTEVLAW
GGFGIVAGLTMWGYRVIATIGKKITELTPTRGFAAEFAAASVVLFASKLGLPISATHTLVGAVMGVGF
ARGLNRVRAETVREIVASWLVTIPVGAVLSIFYTLILTKILAYFM

>VvPHT2.1
MAWNIGANDVANAMGTSVGSGALTLRQAVLTAAVLEFSGALLMGTHVTSTMQKGILVANVFQ
GKDTLLFAGLLSSLAAAGTWLQVASFYGWPVSTTHCIVGSMVGFGLVYGGHGAVFWSSLARVTSS
WVISPLMGAMVSFLVYKCIRRFVYSAPNPGQAAAAAAPLAVFVGVTGISFAAFPLGDSLPSALPKA
LACGVAGAFLVYRIIHKQLGTQLEIVYGVFGYMQILSACFMSFAHGGNDVSNAIGPLAAALSILQG
GTGGSEIVIPLDVLAWGGFGIVAGLMMWGYRVISTIGKKITELTPTRGFAAEFAAASVVLFASKLGLP
ISATHTLVGAVMGVGFARGLNSVRAETVREIVVSWAVTIPVGALLSVFYTWILTKLLAFIP
>KfPHT2.1
MTTSYYCLSFARNVAGAAKLENLKPSYTRHIHLPKIRASKVHPGSLSFVGDELRLKPSTFRHPFASLSS
FAEAGGGEEEDNEHGRGKSSHEAPSKGDEDNSSGMAQAFNISSRTATAISVVIAFAALSFPLFMQS
VSAGLDLKIRALSYATLLFGFYMAWNIGANDVANAMGTSVGSGALTLRQAVLTAAVLEFSGALLM
GRHVTSTMQKGILVAEVFQGKDMLLFAGLLSALAAAGTWLQVASYYGWPVSTTHCIVGAMVGF
GLVYGGTGAVFWSSIARVTSSWVISPALGALVSFLVYKCIRRFVYSAPNPGQAAAASAPIAVFVGVT
GISFASFPLSQTPHIAILQALACGTVGAVLVNRVIRRQLGHLLDQAEPTPPAEKEEAHQTKNIGFLSD
IAGPKGTQLEIVYGVFGYMQILSACFMSFAHGGNDVSNAIGPLSAALSILQGVASGSEIVIPLDVLA
WGGFGIVAGLMMWGYRVIATIGKKITELTPTRGFAAEFAAASVVLFASKLGLPISATHTLVGAVMG
VGFARGFNSVRAETVREIVASWAVTIPVGAFFAVLYTWILTKLLGYIL
>KlPHT2.1
MTTSYYCLSFARNVAGAAKLENLKPSYTRHIHLPKIRASKVHPGSLSFVGDELRLKPSTFRHPFASVS
SFAEAGGGEEEDNEHGRGKSSHEAPSKGDEDNSSGMAQAFNISSRTATAISVVIAFAALSFPLFMQ
SVSAGLDLKIRALSYATLLFGFYMAWNIGANDVANAMGTSVGSGALTLRQAVLTAAVLEFSGALL
MGRHVTSTMQKGILVAEVFQGKDMLLFAGLLSALAAAGTWLQVASYYGWPVSTTHCIVGAMVG
FGLVYGGTGAVFWSSIARVTSSWVISPALGALVSFLVYKCIRRFVYSAPNPGQAAAASAPIAVFVGV
TGISFASFPLSQTPHIAILQALACGTVGAVLVNRVIRRQLGHLLDQAEPTPPAEKEDAHQTKNIGFLS
DIAGPKGTQLEIVYGVFGYMQILSACFMSFAHGGNDVSNAIGPLSAALSILQGVASGSEIVIPLDVL
AWGGFGIVAGLMMWGYRVIATIGKKITELTPTRGFAAEFAAASVVLFASKLGLPISATHTLVGAVM
GVGFARGLNSVRAETVREIVASWAVTIPVGAFFAVLYTWILTKLLGYIL
>OsPHT2.1
MSQSSPFFSVARAHAGAGGRAAAAALLLRHPVAQLPPRIHGLRYYPSAIVSPAKTLNSHLSLPHATI
SSFANADNGSSGQADATESEEEQNGESELSEMAKAFHISPRMAMSISVVIAFAALTVPLAMQSVV
FHGTNKMKALAYLTLLSGFYMAWNIGANDVANAMGTSVGSGALTLRQAVLTAAVLEFSGAFLM
GTHVTSTMQKGILVASVFQGKDSLLFAGLLSSLAAAGTWLQVASSYGWPVSTTHCIVGAMVGFGI
VFGGVNAVFWSSLARVSSSWVISPLMGAAVSFIVYKGIRRFVYSAPNPGQAAAAAAPIAVFTGVTA
ISFAAFPLSKTFSIAILQALACGAIGAVIVNRVIQKQLGDLLSSEAEKIASADKANAQQVGFLSDIAGP
TGAQLQIVYGVFGYMQVLSACFMSFAHGGNDVSNAIGPLAAALSILQGVASSAEIVIPTEVLAWG
GFGIVAGLTMWGYRVIATIGKKITELTPTRGFAAEFAAASVVLFASKLGLPISATHTLVGAVMGVGFA
RGLNRVRAETVREIVASWLVTIPVGAVLSIFYTLLFTKILAYFM
>MePHT2.1
MTSFYCLSSTRNTVTPETVLCNSYLCLSRNRWHRESNFSKTYQLPSKPFLPILRLDNSKLAYPFASISS
YAEAGGEEEQKEGIQIEKHQETVKNHENDLPGMAQAFHISSSTASAISIFIAFAALTLPLFMKSLGQG
LGLKTKLLSYTTLLFGFYMAWNIGANDVANAMGTSVGSGALSLRQAVVTAAILEFAGALLMGTHV
TNTMQKGILVANVFNGKDTLLFAGLLSSLAAAGTWLQVASYYGWPVSTTHCIVGSMVGFGLVYG
GAGAVFWSSLARVTSSWVISPIMGAIVSFLVYKCIRRFVYSARNPGQAAAAAAPIAVFVGVTGISFA
AFPLSKIFPLALAQALACGAAGAFLVSRIIHKQLGHLLVKANSTQPEPQESSSTTHNKTIGFLSDVAG

PKGTQLELVYGVFGYMQVLSACFMSFAHGGNDVSNAIGPLAAALSIIHGGASGTEIVIPTDVLAWG
GFGIVAGLTMWGYRVIATIGKKITELTPTRGFAAEFAAASVVLFASKLGLPISATHTLVGAVMGVGFA
RGLNSVRAETVREIVASWLVTIPAGALFAVIYTWIMTKLLSPML
>MpPHT2.1
MSAVVAALAGGRPHFSTLHDRVSSSASHHDHKTVPRFQRQVPARGLPVLSLSLTTPLSLKVYKSLR
SPLTFVGRNGTRSLTVRSSSIANADDFNVNDGVEKLPEAENMHEGPPSAEDNGTLVRGLSPAHAG
EEKSSGPKGMAEAFHISKGTAFGIITAIAFAAFGVPMFMQSTSALLPVKTRVLSYVTLLFGFYMAWNI
GANDVANAMGTSVGSGALSLRQAVLTAAVLEFGGAFLVGSHVSHTMQKGILRMTVFEGKETLLF
CGMLSSLAAAGTWLQVASFFGWPVSTTHCIIGAMVGFGLLYGGIGAVYWSSLLRVVSSWVVSPF
MGAAVSFLVYMCIRLFVYSAPNPGQAAATVAPIAVFIGVAALSATAFPPRSTLLLTAAQALGLGAV
GAVVINMVIQRQLGSLLGEFCAIPTDDKEEKPLSRSPMKVGGPTGTQLKVVYSIFGYLQVLSACFMS
FAHGANDVANAIGPISAALAILHGTSLSADLGLPAEILAWGGFGIVAGLMVWGYRVIATIGKKITELT
PTRGFAAEFAAASVVVVASRLGLPISATHTLVGAVMGVGFARGLNSVRGEVVREIVASWMVTIPV
GAVLSVVYTLILSKMIPSIF
>MtPHT2.1
MNLNHSCGFSLTKHTRKTSLTSSIYLRNSHIFLSREFLHVKPLPLSPNIAKNLKNCKLTHPFASLSSFAE
AEGEEGSKEEIQLNEHHQHAATENGENNDEVSGMAKAFHISSRTASAITICIVMAALVFPLFMTSL
GQGLALKTKMLSYGTLLFGFYMAWNIGANDVANAMGTSVGSGALSLRQAVLTAAVLEFSGALLM
GTHVTSTMQKGILVANVFQGKDTLLFAGLLSSLAAAGTWLQFASYYGWPVSTTHCIVGAMVGFG
LVYGGAGAVFWGSLARVISSWIFSPLLGAAVSFIVYKCIRRFVYSASNPGQAAAAAAPIAVFLGVTGI
SFAAFPLSKIFPIALSQALACGTVGAFLVNRTIRKQLGHLLDKSNTPDLEPVPQNISFFSDITGPKGTQ
LEIVYGVFGYMQVLSACFMSFAHGGNDVSNAIGPLAGALAILQGAAKGADIVIPIDVLAWGGFGIV
AGLMMWGYRVIATIGKKITELTPTRGFAAEFAAASVVLFASKLGLPISGTHTLVGAVMGVGFARGF
NNVRSETVKEICASWAVTIPVGATLSVIYTWILTKLLSYVL
>MgPHT2.1
MTSSSSSYSSLSSTKNTFLQFRPSQIYLPNYRPSVEFEPHCLRRDFLSLKPQLQSITKPISLSLLTLSSNG
FSRPFAAVSSFAEGDGDGDSAPPKEESAADGEELRGMAQAFHISSGTASAISVCIAVAALVVPLFM
TSLGQGLPFKTRALSYATLLFGFYMAWNIGANDVANAMGTSVGSGALSLRQAVLTAAVLEFSGAL
LMGTHVTSTMQKGILVADVFRGKDTLLFAGLLSSLAAAGTWLQVASYYGWPVSTTHCIVGSMVG
FGLVYGGTGAVFWSSLARVTSSWVVSPLVGALASFLVYKCIRRFVYSAPNPGQAAAAAAPIAVFLG
VTGISFAAFPLSKTLPIAVAQALSAGTAGAFLVDRTIRKQLGHLLANSAEEEADSAGAKSESKNIGFL
SDIAGPTGTNLEIVYGVFGYMQILSACFMSFAHGGNDVSNAIGPLAAALSILQGGMTGGEIVIPND
VLAWGGFGIVAGLMMWGYRVIATIGKKITELTPTRGFAAEFAAASVVLGASKLGLPISATHTLVGAV
MGVGFARGLNSVRAETVREIVTSWAVTIPAGATFAVIYTWILTKVLSYVL
>CsPHT2.1
MPPPFCSSSSRNTVSPEAFLLHKHRSSSSALLLKPNLSLPRSSYSLLSLKNSRLTHSFASISSFAEAEGE
GEEEQNEGLQVQKHHHDEPTKTEAGDDDLPGMAQAFHISSSTASAISICIALAALTLPFFMKSLGQ
GLDIKTKLLSHATLLFGFYMAWNIGANDVANAMGTSVGSGALTLRQAVLTAAVLEFSGALLMGTH
VTSTMQKGILVTSVFQGKDTLLFAGLLSSLAAAGTWLQVASYYGWPVSTTHCIVGSMVGFGLVYG
GAGAVFWSSLARVTSSWVISPILGALVSFLVYKCIRRFVYSAPNPGQAAAAAAPIAVFVGVTGISFA
AFPLSKIFPLALAQALAFGAAGAFLVYRIIHKQLGHLLVKSTSLQPEPKDTNIHNKSIGIFSDIAGPKGT
QLEIVYGVFGYMQVLSACFMSFAHGGNDVSNAIGPLAAALSILHGGASGTKIVIPIDVLAWGGFGI
VGD
>PhPHT2.1

MSQSSRFFSIARAHAGAGGRAAAATLLLRQPVAQLPPIHSLRCFSSARVTDAKTLRSHLSLPRALLS
SFADADDGSGTKAGDAEAREERNGESEMSEMAKAFHISPRMAMSISVMIAFAALTAPLAMRSLVC
HGTFKMSVLAYLTLLSGFYMAWNIGANDVANAMGTSVGSGALTLRQAVLTAAVLEFSGAFLMGT
HVTSTMQKGILVASVFQGKDSLLFAGLLSSLASAGTWLQVASSYGWPVSTTHCIVGAMVGFGLVY
GGVNAVFWSSLAKVSSSWVISPLMGAAVSFLVYKCIRRFVYSAPNPGQAAAAAAPIAVFTGVTAIS
FVAFPLSKVFPIALLQALSCGAVGAIIVTRVIQKQLGELLSSEAEKIASAEKADVQQVGFLSDIAGPTG
AQLKIVYGVFGYMQVLSACFMSFAHGGNDVSNAIGPLAAALSILQGVASSAEIVIPTEVLAWGGFG
IVAGLAMWGYRVIATIGKKITELTPTRGFAAEFAAASVVLFASKLGLPISATHTLVGAVMGVGFARGL
NRVRAETVREIVASWLVTIPVGAVLCIFYTMILTKILAYFM
>PvPHT2.1
MLISSHRFLLPPLSLLLPPLLPLNLISFLLLPPLAPLFCFRHSMSQSSPFFSIARAHAGAGGWTAAAALL
LRQPVAQLPPIQSLRCFSSARVTDAKTLRSHLSLPRASLSSFADADDGSGTKAGDAEAREKRNGESE
MSEMAKAFHIPPRMAMSISVMIAFAALTMPLAMRSLVCHGTFKMSVLAYLTLLSGFYMAWNIGA
NDVANAMGTSVGSGALTLRQAVLTAAVLEFSGAFLMGTHVTSTMQKGILVASVFQGKDSLLFAG
LLSSLAAAGTWLQVASSYGWPVSTTHCIVGAMVGFGLVYGGVNAVFWSSLARVSSSWVISPLMG
AAVSFLVYKCIRRFVYSAPNPGQAAAAAAPIAVFTGVTAISFVAFPLSKVFPIALLQALSCGAVGAIIV
TRVIQKQLGELLSSEAEKIESAEKADVQQVGFLSDIAGPTGAQLQIVYGVFGYMQVLSACFMSFAH
GGNDVSNAIGPLAAALSILQGVASSAEIVIPTEVLAWGGFGIVAGLAMWGYRVIATIGKKITELTPTR
GFAAEFAAASVVLFASKLGLPISATHTLVGAVMGVGFARGLNRVRAETVREIVASWLVTIPVGAVLC
IFYTMILTKILAYFM
>StPHT2.1
MTSSYSLSSIRNSTKFVANNSNLFFPRQRLCVFTNETQFPKKDFLVLKPQTCSFSFLRLKKSSFTHPFA
ALSSFAESDDEKGENFEVKSHQEKAISSENEDELPGMAQAFNISSSTASAVAICIALAALILPFFMKSL
GQGLGLKYKILSYVTILFGFYMAWNIGANDVANAMGTSVGSGALSLRQAVVMAGVLEFSGALLM
GTHVTNTMQKGILVTNVFQGKDTLLFAGLLSSLAAAGTWLQVASYYGLPVSTTHCIVGSMVGFGL
VYGGTGAVFWSSLARVISSWVISPLLGAVVSFLVYKCIRRFVYSARNPGKAAATAAPISVFLGVTGIS
FIALPLSKTLNLALGQAITCGAISAFAVDRIIRGQLGHLLARASSKEPEPEPETTDTKNIGFLSDIAGPK
GTQLKIVYGVFGYMQVLSACFMSFAHGGNDVSNAIGPLASALSILQGGLSAADIVIPNDVLAWGG
FGIVAGLTMWGYRVIATIGKKITELTPTRGFAAEFAAASVVLCASKLGLPISGTHTLVGAVMGVGFA
RGFNSVRAETVREIATSWAVTIPAGATFAVIYTWIFTKLLSYLL
>PvPHT2.1
MNPSCRFASTKHTPFLQNSHITSPFFLRHSHRVKTHLSLPSPTIAPKPSSVNILRFRHLGAAKRFATLS
SFAEGEAGGREEEVQAANGNEVDGLAKAFDISSGTASAISICMALAVLSFPLMMKSLGQGLAVKTK
VLSYATLLFGFYMAWNIGANDVANAMGTSVGSGALTLRQAVLTAAVLEFSGALLMGTHVTNTM
QKGILLANVFNGKDTLLFAGLLSSLAAAGTWLQIASYYGWPVSTTHCIVGAMVGFGLAYGGAGAV
FWGSLARVISSWVVSPLMGAAVSFLVYKCIRRFVYSAPNPGLAAAAAAPIAVFMGVTGISFVAFPLS
KSFPLALAQALVSGTVGAFLVDRIIRKQLGHLLVKSNTPKPESKEDTVHHNIGFLDDVAGPKGAQLE
IVYGVFGYMQVLSACFMSFAHGGNDVSNAIGPLAGALAILQGGAAGAEIVIPIDVLAWGGFGIVA
GLMMWGYRVIATIGKKITELTPTRGFAAEFAAASVVLFASKLGLPISATHTLVGAVMGVGFARGFNS
VRSETVKEIVASWVVTIPVGAVLSVIYTWILTRILSYIL
>PtPHT2.1
MVGFGLVYGGRGAVFWSSLARVTSSWVISPLMGAMVSFLVYKFIRRFVYSAPNPGQAAAAAAPIA
VFLGVTGISFAAFPLSEIFPLALAQALACGTTGAFLVDRIIRKKLGHLLVKASSTQPELKENAIHSKNIG
FLSDFAGPKGTQLEIVYGVFGYMQILSACFMSFAHGGNDVSNAIGPLAAALSILHGGASGTDIVIPI

DVLAWGGFGIVAGLMMWGYRVIATIGKKITELTPTRGFAAEFAAASVVLVASKLGLPISATHTLVGA
VMGVGFARGLNSVRAETVREIVVSWAVTIPAGAIFAVFYTWILTKLLSYIL
>PtPHT2.2
MTLFYTLSSSTRNIISPETHVLHNSHFYLPRNRSSFFSNETTLFKRETLPFKPQQQQPTGSFPPILRLKN
SKLAHPFASISSYAEAGGEEEKNEGVQIEEHQETVKKKENDSPGMAQAFDISSRTASAISILIAFAALS
LPLFMKTLGQGLDLKTKFLSYVTLLFGFYMAWNIGANDVANAMGTSVGSGALTIRQAVLTAAVLE
FSGALLMGTHVTGTMQKGILVANVFQGKDTLLFAGLLSSLAAAGTWLQVASYYGWPVSTTHCIV
GSMVGFGLVYGGPGAVFWSSLARVTSSWVISPLMGAMVSFLVYKFIRRFVYSAPNPGLAAAAAAP
IAVFLGVTGISFAAFPLSKVFPLALAQALASGAVGAFLVDRIIRKQLGHLLVKASSSQPEPKENAIHSK
NIGLLSDFAGPKGTQLEIVYGVFGYMQILSACFMSFAHGGNDVSNAIGPLAAALSILHGGASGTEIV
IPMDVLAWGGFGIVAGLMMWGYRVIATIGKKITELTPTRGFAAEFAAASVVLVASKLGLPISATHTL
VGAVMGVGFARGLNSVRAETVREIVVSWAVTIPVGAIFAVFYTWILTKLLSYIL
>PpPHT2.1
MGSMTRLGQGITPLESNVKSLHIRISRNGVHGFRTPPHLYRFGRQKCKSIVPIQIEPNNDGILKPEIQ
WCGIKPRRSLGCRASSADAGTGDLHGNIGNDFRSEVGIVKEEATNGGPKGMAEAFDISPRTALGIT
AVIAISVLVLPMYMPSSGVGMTLRTRGLSYLTLLLGFYMAWNIGANDVANAMGTSVGSGALTLRQ
AVLTAAVLEFSGAFMVGSHVSHTMQSGILTPSVFAGKETLLFCGMLSSLGAAGTWLQVASYFGWP
VSTTHCIIGAMVGFGLVYGGVGAVYWKSLFRVVSSWVVSPLLGAFVSFLVYKCIRRFVYRAPNPGQ
AAATAAPLAVFLGVAALSFTVLPTQGVGLMAGGKALGLGLLGAVIINIVIRRQLGELLGAYCELPSEE
EQQGKASRDEGPKGTQLKIVYSVFGYLQVLSACFMSFAHGANDVANAIGPISAALAILHKTGVASG
GPPSMPVDVLAWGGFGIVAGLVVWGYRVIATIGQKITELTPTRGFAAEFAAATVVVLASRLGLPISA
THTLVGAVMGVGFARGLNSVRTDVVREIVASWVVTIPVGAALSVAYTFIFIKLIPSLV
>PePHT2.1
MTPSYCLSSARNTITPEAFLLHNSHLYLPKHRSFSFSTETHFPRKETNFLRPQTPLPKSFLPILRLNNSK
ITQPFASLSSFAEAEGEKQQNQDAKAEEHHENAKTEDQAESPGMAQAFHISSRTASAISICIVFAAL
SLPFFMKSLWQGMALKTKMLSYATLLFGFYMAWNIGANDVANAMGTSVGSGALTLRQAVVTAA
VLEFSGAFFMGTHVTSTMQKGILVANVFQGKDTLLFAGLLSSLAAAGTWLQVASYYGWPVSTTHC
IVGSMVGFGLVYGGAGAVFWSSLARVISSWVVSPFMGALVSFLVYKCIRRFVYSAPNPGQAAAAA
APIAVFVGVSGISFAAFPLGENLALALARALACGIAGAVLVYRIIRKQLGHLLLKSTSSEAEQKEGTIHP
KNIGFLSDIAGPTGTQLEIVYGVFGYMQILSACFMSFAHGGNDVSNAIGPLAAALSILHGGASGAEI
VIPTDVLAWGGFGIVAGLTMWGYRVIATIGKKITELTPTRGFAAEFAAASVVLFASKLGLPISATHTL
VGAVMGVGFARGLNRVRAETVREIVASWAVTIPAGAFFSVVYTWILTKLLSYIL
>SpPHT2.1
MTLSYTLSSSTGNIISPDSRVLHSSHFNPPRNRFSFFSSETSLFKRETFPFKPQQQQVPKSFLPILRLDS
SKPAHPFASLSSYAEAGGEEDEGIQTEKQQETVKKEDVSPGMAQAFDISSRTASAISILIAFAALSVPL
CMKTLGMGLDLKTKFLSYVTLLFGFYMAWNIGANDVANAMGTSVGSGALTIRQAVLTAAVLEFSG
ALLMGTHVTSTMQNGILVANVFQGKETLLFAGLLSSLAAAGTWLQVASYFGWPVSTTHCIIGSMV
GFGLVYGGPGAVFWSSLARVTSSWVISPLMGAMVSFLVYKFIRRFVYSAPNPGQAAATAAPMAVF
LGVTGISFAAFPLSKVFHLALAQAVASGAVGAFLVDRIIRKQLGHLLVKASSSQSEPKENAIHSKNIG
LLSDFAGPKGTQLEIVYGVFGYMQILSACFMSFAHGGNDVSNAIGPLAAALSILHGGASGAEIVIPV
DVLAWGGFGIVAGLMMWGYRVIATIGKKITELTPTRGFAAEFAAASVVLVASKLGLPISATHTLVGA
VMGVGFARGLNSVRAETVREIVVSWVVTIPVGAIFAVFYTWIMTKLLSYIL
>SiPHT2.1
MSQSSPFFSIARAHAGAGGRAAAAALLLRQPVAQLPTNIHSLRCFPSARVTHAKTLRSHLNLPRAT

LSSFADADDGSKTKPDEAEAGEEQNGECEMSEMAKAFHISPRMAMSISMTTAFAALTVPLAMRSL
VCRGTFKMSVLAYLTLLSGFYMAWNIGANDVANAMGTSVGSGALTLRQAVLTAAVLEFSGAFLM
GTHVTSTMQKGILVASVFQGKDSLLFAGLLSSLAAAGTWLQVASSYGWPVSTTHCIVGAMVGFGL
VYGGVNAVFWSSLARVSSSWVISPLMGAAVSFLVYKCIRRFVYSAPNPGQAAAAAAPIAVFTGVT
AISFAAFPLSKVFPIALLQALSCGAIGAIIVTRVIQKQLGELLSSEAEKIASADKSNVQQVGFLSDIAGP
TGAQLQIVYGVFGYLQVLSACFMSFAHGGNDVSNAIGPLAAALSILQGVASSAEIVIPTEVLAWGG
FGIVAGLTMWGYRVIATIGKKITELTPTRGFAAEFAAASVVLFASKLGLPISATHTLVGAVMGVGFSR
GLNRVRVETVREIVVSWLVTIPAGAVLCVLYTTILTKILAYFM

>SvPHT2.1

MSQSSPFFSIARAHAGAGGRAAAAALLLRQPVAQLPTNIHSLRCFPSARVTHAKTLRSHLNLPRAT
LSSFADADDGSKTKPDEAEAGEEQNGECEMSEMAKAFHISPRMAMSISMTTAFAALTVPLAMRSL
VCRGTFKMSVLAYLTLLSGFYMAWNIGANDVANAMGTSVGSGALTLRQAVLTAAVLEFSGAFLM
GTHVTSTMQKGILVASVFQGKDSLLFAGLLSSLAAAGTWLQVASSYGWPVSTTHCIVGAMVGFGL
VYGGVNAVFWSSLARVSSSWVISPLMGAAVSFLVYKCIRRFVYSAPNPGQAAAAAAPIAVFTGVT
AISFAAFPLSKVFPIALLQALSCGAIGAIIVTRVIQKQLGELLSSEAEKIASADKSNVQQVGFLSDIAGP
TGAQLQIVYGVFGYLQVLSACFMSFAHGGNDVSNAIGPLAAALSILQGVASSAEIVIPTEVLAWGG
FGIVAGLTMWGYRVIATIGKKITELTPTRGFAAEFAAASVVLFASKLGLPISATHTLVGAVMGVGFSR
GLNRVRVETVREIVVSWLVTIPAGAVLCVLYTTILTKILAYFM

>SbPHT2.1

MSQSSPFFSIARVHAGAGGRAAAAALLLRRSISQLPHSIHGLRCFSSARVTPAKTLQSHLSLPRATLS
SFADAEDGSSAEGSDKKAGEEQIGECEMSEMAKAFHISPRMAMSISVMIAFAALTVPLAMRSLVC
HGTFKMNALAYFTLLSGFYMAWNIGANDVANAMGTSVGSGALTLRQAVVTAAVLEFSGAFLMG
THVTSTMQKGILVASVFQGKDSLLFAGLISSLAAAGTWLQVASSYGWPVSTTHCIVGAMVGFGLV
YGGVNAVFWSSLARVSSSWVISPLMGAAVSFLVYKCIRRFVYSAPNPGQAAAAAAPIAVFAGVTAI
SFAAFPLSKVFSIAILQAVSSGAIGAIIVSRVIKKQLGDLLSSEAEKIASTEKSNVQQAGLLSDIAGPTG
AQLQIVYSVFGYMQVLSACFMSFAHGGNDVSNAIGPLAAALSILQGVASSAEIVIPTEVLAWGGFG
IVAGLTMWGYRVIATIGKKITELTPTRGFAAEFAAASVVLFASKLGLPISATHTLVGAVMGVGFARGL
NRVRAETVRDIVASWLVTIPVGAVLSIFYTLILTKILAYLM

>SPHT2.2

MTSSYSLCSIRNSTKFVANNSNLFFPRQRLCVFTNETQFPKKDFLVLKPQTCCFSFLRLKKSSFKHPF
AALSSFAESDDEKGENFEIKSHQENTISSEDEDELPGMAQAFHISSRTASAISICIALAALILPFFMKSL
GQGLGFKYKILSYVTILFGFYMAWNIGANDVANAMGTSVGSGALSLRQAVVMAGVLEFSGALLM
GTHVTNTMQKGILVANVFQGKDTLLFAGLLSSLAAAGTWLQVASYYGLPVSTTHCIVGSMVGFGL
VYGGTGAVFWSSLARVISSWVISPLLGAVVSFLVYKCIRRFVYSARNPGKAAATAAPISVFLGVTGIS
FVALPLSKTLNLALGQAITCGAISAFAVDRIIRGQLGHLLAKASSKEPETTDTKNIGFLSDIAGPKGTQ
LKIVYGVFGYMQVLSACFMSFAHGGNDVSNAIGPLAAALSILQGGLSAADIVIPNDVLAWGGFGIV
AGLTMWGYRVIATIGKKITELTPTRGFAAEFAAASVVLCASKLGLPISGTHTLVGAVMGVGFARGFN
SVRAETVREIATSWAVTIPAGATFAVIYTWIFTKLLSYLL

>SfPHT2.1

MLKLGRSCCSSSLEAAATDHGLLLSRPALGRLHRRIFCWHVPAVGRIDTNPSISSRCMQQPWHISS
RSSRPRLTLSLPKCGNEQDSNTSVCSASLADAGEFHEEIESFQRLKTVKKVSESKGMDGMAEAFHIT
PRLAVGITAAIGLAALVVPMYMPSSAPGMVFKTRALSYITLLFGFYMAWNIGANDVANAMGTSVG
SGALSLRQAVLTAAVLEFSGAFMVGSHVSHTMQSGILTAGIFNGKDSLLFCGMLSSLGAAGTWLQ
VASYFGWPVSTTHCIIGAMVGFGLIYGGIGAVYWKSLARVVASWVVSPFLGAVVAFVVYKCIRTFV

YSAPNPGQAAATAAPIAVFLGAAALSFTVFPSKASVGMAAAQALGFGFLAAIVINIVIRRQLGDLLG
LFYELPAEGQVDEKASKQPVKFMGKVEGPKGTQLRIVYSVFGYLQVLSACFMSFAHGANDVANAI
GPISAALAILHGTGLNTGAQPSMPIDVLAWGGFGIVAGLLVWGYRVIATIGEKITELTPTRGFAAEFA
AATVVVLASRMGLPISATHTLVGAVMGVGFARGLNSVRGEVVREIVASWMVTIPVGAFLSVVYTFI
LIRLIPSLV
>TcPHT2.1
MTPFSCLPSARNTTSPEAFLLRNPHLHVPKYRYNETQLPKKENLMWMPFQVPTRPYRPILSLTNSKL
TQPSASISSFAEAEGEKEQKEGIQLETHDQEDAKTNDDDLPAMAQAFHISSRTASAIAIFIAFAALSL
PFFMKSLGQGVSLKTKFLSYATLLFGFYMAWNIGANDVANAMGTSVGSGALTLRQAVLTAAILEFS
GALLMGTHVTSTMQKGILVANVFQGKDSLLFAGLLSSLAAAGTWLQVASYYGWPVSTTHCIVGS
MVGFGLVYGGAGAVFWSSLARVTSSWIVSPIMGAMVSFLVYKCIRRFVYSAHNPGQAAAAAAPI
AVFVGVTGISFAAFPLSKSYAIGLAQALGCGAAGALLVHKIINKQLGHLLVKSISSSHSEPQENTTQN
KNLGLLSDIAGPKGTQLEIVYGVFGYMQVLSACFMSFAHGGNDVSNAIGPLAAALSILQGGSGNEI
VIPNDVLAWGGFGIVAGLMMWGYRVIATIGKKITELTPTRGFAAEFAAASVVLFASKLGLPISATHTL
VGAVMGVGFARGLNRVRAETVREIVTSWVITIPVGASLAVLYTWILTKLLPNIS
>EsPHT2.1
MTLPYRFSSVRNHSLLLRTSHLCTPRSALGCFSPKESPFFKKTSTIFLSPHKHTSLPLKLVCPLASFSSY
ADSEGDEKHHGDQQIQNSNESSTNSTESDGKGNAEATGDFSGMAQAFNISSNTARAISIAIAFSA
LSFPLFMKSLGQGLALKTKLLSYATLLFGFYMAWNIGANDVANAMGTSVGSGALTIRQAVMTAAV
LEFSGALLMGTHVTSTMQKGILMANVFQGKDMLLFAGLLSSLAAAGTWLQVASYYGWPVSTTHC
IVGSMVGFGLVYGGAGAVFWSSLAKVASSWVISPVMGALVSFLVYKCIRRFVYSAPNPGQAAAAA
APVAVFVGIASISSAAFPLSKTFPIAISQALACGAAGAIVFDRIIRNKLGHLLAKTKSPETSQNQPKSIG
FLSDIAGPTGTQLEIVYGIFGYMQVLSACFMSFAHGGNDVSNAIGPLAAALSILQKGAAAGGGEIVI
PMDVLAWGGFGIVAGLTMWGYRVIATIGKKITELTPTRGFAAEFAAASVVLFASKLGLPISATHTLV
GAVMGVGFARGLNSVRAETVKEIVASWLVTIPVGATLAVIYTWVFTKILSFVL
>TpPHT2.1
MNLNHSCGFSLTKQTTKSISSFYLRNSQVFLFKNTSPLFLREFLHVKTLPFSTNITKSLRNCKLTHPFA
SLSSFAEAEGEEGSKEEIEHHNATKNGEKENNNGEVSGMAKAFNISSRTASAITICIAMAALVFPLF
MTSLGQGLTLKTKVLSYATLLFGLYMAWNIGANDVANAMGTSVGSGALTLRQAVLTAAVLEFSG
ALLMGTHVTSTMQKGILVANVFQGKDSLLFAGLLSSLAAAGTWLQFASYYGWPVSTTHCIVGAM
VGFGLVYGGAGAVFWGSLARVISSWIFSPLLGAAVSFLVYKCIRRFVYSASNPGQAAAASAPIAVFL
GVTGISFSAFPLSKTFPIALSQALACGTVGAFLVNRTIRKQLGHLLDKPNTTQSEPKDDDTAHQNIG
FFSDITGPKGAQLEIVYGVFGYMQVLSACFMSFAHGGNDVSNAIGPLAAALAILQGAAKGAEIVIPI
DVLAWGGFGIVAGLMMWGYRVIATIGKKITELTPTRGFAAEFAAASVVLFASKLGLPISATHTLVGA
VMGVGFARGLNSVRSETVKEICASWAVTIPVGATLSVIYTWILTKLLSNVF
>ZaPHT2.1
MSQSSPLFSVARAHAGAGGRAVAAALLLRRRPIAQLPHSIHGLRCLSPARATPSKTLLSLPRATLSSF
ADPDGDGGSSAKASDGGAKAGAGGEEQVGECEMSEMARAFHISPRVATSISVTIAFAALTVPLAM
RSLVCHGTFRMNALAYFTLLSGFYMAWNIGANDVANAMGTSVGSGALTLRQAVLTAAVLEFSGA
FLMGTHVTSTMQKGILVASAFQGKDSLLFAGLISSLAAAGTWLQVASSYGWPVSTTHCIVGAMVG
FGLVYGGVDAVFWSSLARVSSSWVISPLMGAAVSFLVYKCIRRFVYSAPNPGQAAAAAAPIAVFTG
VTAISFAAFPLSKVFSIAILQAVSSGAIGAIIVSRVIQKQLGDLLSSEAEKIASAEKSSVQQAGFLSDIAG
PTGAQLQIVYGVFGYMQVLSACFMSFAHGGNDVSNAIGPLAAALSILQGVASSAEIVIPTEVLAW
GGFGIVAGLTMWGYRVIATIGKKITELTPTRGFAAEFAAASVVLFASKLGLPISATHTLVGAVMGVGF

ARGLNRVRAETVREIVASWLVTIPVGAVLSIFYTLILTKILAYFM
>CaPHT2.1
MNLNHSCGFSLTKHTRKTSLTSSIYLRNSHIFLSREFLHVKPLPLSPNIAKNLKNCKLTHPFASLSSFAE
AEGEEGSKEEIQLNEHHQHAATENGENNDEVSGMAKAFHISSRTASAITICIVMAALVFPLFMTSL
GQGLALKTKMLSYGTLLFGFYMAWNIGANDVANAMGTSVGSGALSLRQAVLTAAVLEFSGALLM
GTHVTSTMQKGILVANVFQGKDTLLFAGLLSSLAAAGTWLQFASYYGWPVSTTHCIVGAMVGFG
LVYGGAGAVFWGSLARVISSWIFSPLLGAAVSFIVYKCIRRFVYSASNPGQAAAAAAPIAVFLGVTGI
SFAAFPLSKIFPIALSQALACGTVGAFLVNRTIRKQLGHLLDKSNTPDLEPVPQNISFFSDITGPKGTQ
LEIVYGVFGYMQVLSACFMSFAHGGNDVSNAIGPLAGALAILQGAAKGADIVIPIDVLAWGGFGIV
AGLMMWGYRVIATIGKKITELTPTRGFAAEFAAASVVLFASKLGLPISGTHTLVGAVMGVGFARGF
NNVRSETVKEICASWAVTIPVGATLSVIYTWILTKLLSYVL
>SmPHT2.1
MSQSSPFFSIARAHAGAGGRAAAAALLLRCPAAQLPPTIHCPRYFRLAKVSPAKTLSSHLVLPRATL
SSFADADDGSSANSDASGERSGGSELSEMAKAFHISPRMAMSISVVIAFAALTVPLAMRSLLFHGT
TKMKVLAYLTLLSGFYMAWNIGANDVANAMGTSVGSGALTLRQAVLTAAVLEFSGAFLMGTHVT
STMQKGILVTSVFQGNDSLLFAGLLSSLAAAGTWLQVASSYGWPVSTTHCIVGAMVGFGLVYGG
VNAVFWSSLARVSSSWLISPLMGAAVSFLVYKCIRRFVYSAPNPGQAAAASAPIAVFTGVTAISFAA
FPLSRTFSIAVLQALVCGAIGAVFVSRAIKKQLGDLLSSEAEKIATADNTDVQQGGFDVAGPRGAQL
QIVYGVFGYMQVLSACFMSFAHGGNDVSNAIGPLAAALSLLQGVASSAEIVIPTEVLAWGGFGIVA
GLTMWGYRVIATNWQEKSPELTPTRGFAAEFAAASVVLFASKLGLPISATHTLVGAVMGVGFARG
LNRVRAETVREIVVSWVVTIPVGALLSVFYTLILTKILKYFM
>TaPHT2.1
MAKDQLQVLNALDVAKTQLYHFTAIVIAGMGFFTDAYDLFCISLVTKLLGRIYYHHDGAPKPGTLP
PNVSAAVNGVAFCGTLAGQLFFGWLGDKMGRKRVYGMTLMMMVICSIASGLSFGHTPKSVMTT
LCFFRFWLGFGIGGDYPLSATIMSEYANKKTRGAFIAAVFAMQGFGILAGGMVAIIVSAAFKGAFPA
QTYQTDPLGSTVSQADFVWRIILMFGAIPAAMTYYWRMKMPETARYTALVAKNLKQAANDMSK
VLQVDIEEEQEKVENVSQNTRNEFGLFSKEFLRRHGLHLLGTASTWFLLDIAFYSQNLFQKDIFSAIG
WIPPAQTMNALEEVYKIARAQTLIALCSTVPGYWFTVFFIDKIGRFAIQLMGFFFMTVFMFALAIPYH
HWTLKDNRIGFVIMYSLTFFFANFGPNATTFVVPAEIFPARLRSTCHGISAAAGKAGAMIGAFGFLY
AAQPTDRKKADAGYPAGIGVRNSLIVLGCVNFLGMVFTFLVPESKGKSLEEMSRENEGEEESGTEM
KNSGRTVPV
>PHO84
MSSVNKDTIHVAERSLHKEHLTEGGNMAFHNHLNDFAHIEDPLERRRLALESIDDEGFGWQQVKT
ISIAGVGFLTDSYDIFAINLGITMMSYVYWHGSMPGPSQTLLKVSTSVGTVIGQFGFGTLADIVGRK
RIYGMELIIMIVCTILQTTVAHSPAINFVAVLTFYRIVMGIGIGGDYPLSSIITSEFATTKWRGAIMGAV
FANQAWGQISGGIIALILVAAYKGELEYANSGAECDARCQKACDQMWRILIGLGTVLGLACLYFRL
TIPESPRYQLDVNAKLELAAAAQEQDGEKKIHDTSDEDMAINGLERASTAVESLDNHPPKASFKDF
CRHFGQWKYGKILLGTAGSWFTLDVAFYGLSLNSAVILQTIGYAGSKNVYKKLYDTAVGNLILICAG
SLPGYWVSVFTVDIIGRKPIQLAGFIILTALFCVIGFAYHKLGDHGLLALYVICQFFQNFGPNTTTFIVP
GECFPTRYR
>PHO89
MALHQFDYIFAIAMLFAFLDAFNIGANDVANSFASSISSRSLKYWQAMVLAGLCEFLGAVLAGARV
SGTIKNNIIDSSIFTNDPAVLMLTMTSALIGSSCWLTFATAIGMPVSTTHSIVGGTIGAGIAAGGANG
VVWGWSGVSQIIASWFIAPILAGAIAAIVFSISRFSVLEVKSLERSIKNALLLVGVLVFATFSILTMLIVW

KGSPNLHLDDLSETETAVSIVLTGAIASIVYFIFFYPFYRRKVLDQDWTLKLIDIFRGPSFYFKSTDDIPP
MPEGHQLTIDYYEGRRNLGTTVSVEDEENKAASNSNDSVKNKEDIQEVDLVRTETEPETKLSTKQY
WWSLLKQGPKKWPLLFWLVISHGWTQDVIHAQVNDRDMLSGDLKGMYERSKFYDNRVEYIYSV
LQAITAATMSFAHGANDVANATGPLSAVYVIWKTNTIGAKSEVPVWVLAYGGVALVIGCWTYGY
NIIKNLGNKMILQSPSRGFSIELAVAITTVMATQLGIPTSTTQIAVGGIVAVGLCNKDLKSVNWRMV
AWCYSGWFLTLPIAGLIAGIINGIILNAPRFGVEYQMT
