# Supplementary material for: Effects of Soybean Phosphate Transporter Gene GmPHT2 on Pi Transport and Plant Growth under Limited Pi Supply Condition
Source: Int J Mol Sci. 2023 Jul 5;24(13):11115. doi: 10.3390/ijms241311115 (PMC10342517; doi:10.3390/ijms241311115)
Supplement: Supplementary file 1 [file ijms-24-11115-s001.zip › Table S3.pdf]

Table S3. The gene chips expression analysis of *GmPHT2;1* and *GmPHT2;2*.

ID	Nodule	Leaves	Root	Flower	Green Pods	SAM	Root	48 HAI stripped root	Root hairs 12 HAI	Root hairs 12 HAI mock	Root hairs 24 HAI	Root hairs 24 HAI mock	Root hairs 48 HAI	Root hairs 48 HAI mock
GmPHT2;1	0	56.46	0.79	24.61	34.85	30.9	0.31	0.78	2.42	3.18	4.44	1.38	1.54	0.73
GmPHT2;2	0.53	97.66	0.79	32.62	32.11	32.68	0	4.15	5.24	5.3	4.72	4.7	2.15	1.47
