# Supplementary material for: Effects of Soybean Phosphate Transporter Gene GmPHT2 on Pi Transport and Plant Growth under Limited Pi Supply Condition
Source: Int J Mol Sci. 2023 Jul 5;24(13):11115. doi: 10.3390/ijms241311115 (PMC10342517; doi:10.3390/ijms241311115)
Supplement: Supplementary file 1 [file ijms-24-11115-s001.zip › Table S4.pdf]

Table S4. Primers used for GmPHT2 PCR analysis.

ID	Prime
PHT2;1-pYES2F	CGGAATTCATGAATCCCTCTTGTCGCTT
PHT2;1-pYES2R	CATGCATGCTAAAATATAAGACAAAATCTTAGTCAAGA
PHT2;1-p1302SNBF	GGGGTACCATGAATCCCTCTTGTCGCTT
PHT2;1-p1302SNBR	GCGTCGACTAAAATATAAGACAAAATCTTAGTCAAGA
PHT2;1-pPSNF	GCGTCGACATGAATCCCTCTTGTCGCTT
PHT2;1-pPSNR	GGGGTACCTAAAATATAAGACAAAATCTTAGTCAAGA
PHT2;2-pYES2F	CGGGATCCATGAATCCCTCTTGTCGCTT
PHT2;2-pYES2R	CATGCATGCTAAAATATAGGACAAAATCTTAGTCAAG
PHT2;2-p1302SNBF	CGGGATCCATGAATCCCTCTTGTCGCTT
PHT2;2-p1302SNBR	GGGGTACCTAAAATATAGGACAAAATCTTAGTCAAG
qPHT2;1-F	CTGAGGGAGAACAACAAGAGAA
qPHT2;1-R	AAGCCGTTCGAGACGATATG
qPHT2;2-F	AGGGTGCTCAACTGGAAATAG
qPHT2;2-R	CGGCACCACCTTGAAGAATA
